# Within‐Plant Leaf Maturity and Trichome Density Variation Shape the Elemental Composition Divergence in Desert Plant Sand Rice (*Agriophyllum squarrosum*)

**DOI:** 10.1002/ece3.71542

**Published:** 2025-06-12

**Authors:** Ruilan Ran, Xiaoyun Cui, Xin Zhao, Yingxue Zhao, Caixia Zhang, Pengshan Zhao

**Affiliations:** ^1^ State Key Laboratory of Ecological Safety and Sustainable Development in Arid Lands Northwest Institute of Eco‐Environment and Resources, Chinese Academy of Sciences Lanzhou People's Republic of China; ^2^ Key Laboratory of Stress Physiology and Ecology in Cold and Arid Regions, Gansu Province Northwest Institute of Eco‐Environment and Resources, Chinese Academy of Sciences Lanzhou People's Republic of China; ^3^ University of Chinese Academy of Sciences Beijing People's Republic of China; ^4^ Institutional Center for Shared Technologies and Facilities of NIEER, CAS Lanzhou People's Republic of China

**Keywords:** *Agriophyllum squarrosum*, leaf age, mineral composition, subindividual variation, trichomes

## Abstract

Subindividual variation in leaf elemental composition, driven by trichome density and leaf maturity, is critical for plant adaptation but poorly understood in desert species. Here, we reveal that sand rice (*Agriophyllum squarrosum*), a potential future food crop, exhibits obvious declines in leaf trichome density across developmental stages, significantly influencing the redistribution of 33 mineral elements. The concentrations of these elements varied between genotypes (wild type: Shapotou, SPT; *trichomeless1* mutant: *astcl1*) and/or across leaf ages (top, middle, bottom). Sixteen key elements (e.g., S, Ca, Fe, Mn) were identified as primary factors of elemental variance. Our findings reveal a specific subindividual mineral composition of sand rice influenced by trichome density and leaf maturity, providing insights into adaptive trends in leaf nutrient traits and enhancing our understanding of the strategies plants employ to thrive in barren sandy environments.

## Graphical abstract

The density and adhesion of trichomes vary across different developmental stages. The mineral element profiles also change between the two genotypes (wild‐type SPT and *astcl1* mutant) and across different leaf ages, with 16 key elements identified as the main factors influencing these changes.

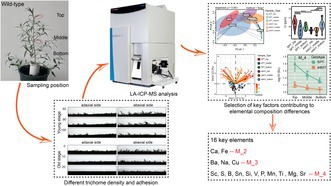

**Abbreviations**
Ag: silver
Al: aluminum
As: arsenic
B: boron
Ba: barium
Be: beryllium
Bi: bismuth
Br: bromine
Ca: calcium
Cl: chlorine
Co: cobalt
Cr: chromium
Cs: cesium
Cu: copper
Fe: iron
Ga: gallium
Ge: germanium
Hg: mercury
I: iodine
In: indium
K: Potassium
Li: lithium
Mg: magnesium
Mn: manganese
Mo: molybdenum
Na: sodium
Nb: niobium
Ni: nickel
P: phosphorus
Pb: lead
Pd: palladium
Rb: rubidium
Rh: rhodium
Ru: ruthenium
S: sulfur
Sb: antimony
Sc: scandium
Se: selenium
Si: silicon
Sn: tin
Sr: strontium
Te: tellurium
Ti: titanium
Tl: thallium
V: vanadium
Y: yttrium
Zn: zinc

## 1 Introduction

Functional trait variability can occur at different hierarchical levels, including biome, landscape, community, species, populations, and individuals (Kissling et al. 2018; Mayor et al. 2023; Sobral 2023). In plants, subindividual variation arises from ontogenetic changes, organ‐specific responses to microenvironmental variations, and developmental instability (Sobral et al. 2013; Sobral 2023). Subindividual variation often exceeds differences among individuals or populations, significantly impacting population persistence and community assembly (Herrera 2017; Alonso et al. 2018). For example, it drives selection for flowering time, optimizes resource use (e.g., water, nitrogen), and enhances adaptation to environmental uncertainty (Austen et al. 2015; Herrera et al. 2015; Herrera 2017; Alonso et al. 2018; Herrera et al. 2021; Møller et al. 2023; Sobral 2023). These effects underscore its critical role in ecosystem functioning.

Leaf traits such as specific leaf area (SLA), leaf dry mass per unit area (LMA), and leaf size are widely used to predict ecosystem processes. However, recent studies challenge their utility. For example, Firn et al. (2019) reported that leaf nutrient concentrations (e.g., nitrogen, phosphorus and potassium) outperform SLA in predicting grassland responses to environmental stresses. Similarly, Furey and Tilman (2023) found that tissue‐level elemental composition (e.g., % Ca, % N, and % K) correlates more strongly with ecosystem functioning than morphological and physiological traits. These findings highlight the potential of elemental variability to bridge biodiversity and ecological processes (El‐Sabaawi et al. 2023). Despite this progress, subindividual variation in elemental traits remains understudied. Such variability, driven by ontogeny, microenvironmental responses, or developmental instability, could refine predictions of plant adaptation and ecosystem resilience.

Leaf traits, including nutrient concentrations, SLA, and LMA, vary across developmental stages (Pasche et al. 2002; Herrera et al. 2015; Dayrell et al. 2018; Ji et al. 2021; Turfan et al. 2020). Plants dynamically reallocate minerals between leaves via phloem transport, optimizing resource use (Watanabe et al. 2016). For instance, mobile elements like K, P, and Mg are recycled from older to younger leaves, while immobile elements such as Ca and Mn remain in older leaves to support structural roles (Marschner 2012; Watanabe et al. 2016; Marler and Krishnapillai 2019). Zn concentrations increase in old and mature grapefruit leaves, likely due to restricted phloem mobility (Tian et al. 2014). Similar patterns occur in hyperaccumulator plants, where leaf age influences Mn, Ni, and Cd retention (Boyd and Jaffré 2009; Lu et al. 2013; Losfeld et al. 2015; Hu et al. 2019). These dynamics underscore the need to study subindividual variation in elemental distribution. Understanding age‐specific nutrient allocation is critical for predicting plant responses to environmental stressors.

Trichomes, hair‐like epidermal structures, vary morphologically (single/multicellular; glandular/non‐glandular) and functionally (Werker 2000; Wang et al. 2021; Dong et al. 2023). Their density often declines with leaf age, peaking in young leaves (Bourland et al. 2003; Shahzad et al. 2021). Beyond the protective functions, for example, microbial invasions, UV damage, dehydration, high salinity, thermal stress, heavy metal toxicity (Agarie et al. 2007; Hauser 2014; Bickford 2016; Liu et al. 2022), trichomes regulate foliar nutrient absorption and distribution. Mineral nutrients such as Mg, Ca, K, and Fe localize to trichome bases in *Alyssum* species (Marmiroli et al. 2004). Cucurbitaceae trichomes exhibit species‐specific Si distribution (Abe 2019). Non‐glandular trichomes sequester Cd in *Arabidopsis* *thaliana* and Zn in soybean, tomato, and sunflower (Zhao et al. 2000; Sarret et al. 2009; Li et al. 2018; Li et al. 2019; Ricachenevsky et al. 2021). Trichome‐less mutants of *Mesembryanthemum crystallinum show* reduced Na^+^/Cl^−^ accumulation and salt sensitivity (Agarie et al. 2007), highlighting their role in ion homeostasis. These studies collectively link trichome presence to nutrient distribution during leaf development.

Desert plants in arid and semi‐arid regions employ diverse strategies to effectively overcome nutrient scarcity caused by drought, salinity, extreme temperatures, and low soil fertility (Marschner 2012). Essential elements (such as N, P, K, Ca, and Mg) critical for photosynthesis, osmotic adjustment, enzymatic activity, and water‐use efficiency are significantly influenced by drought and salinity stress (Mengel and Kirkby 2001; He et al. 2016; Hafez et al. 2021). Under water deficit, plants allocate more C to leaves while reducing N and P concentrations as a strategy to optimize resource use (Zhang, Luo, et al. 2019). Nutrient resorption during leaf senescence further enhances efficiency (Mediavilla et al. 2014; Wang et al. 2014; Zhang, Zhou, et al. 2019). For example, desert shrubs resorb N, P, K, Cu, Mg, and Mn from aging leaves, though drought impairs the resorption of N, P, K, and Cu (Killingbeck 1993; Zhang, Zhou, et al. 2019). While previous studies on nutrient allocation in different desert shrub species have largely focused on certain elements stoichiometry, particularly N, P, K, Fe, Zn, and Mg (Romney et al. 1980; Zhang et al. 2013; Zhang, Zhou, et al. 2019; Zheng et al. 2023), it is essential to consider these variations within the framework of subindividual trait variability.

Sand rice (*Agriophyllum squarrosum*), a member of the Amaranthaceae *sensu lato* family, thrives in deserts and arid regions across Central Asia, Mongolia, Russia, and northern China (Chen et al. 2014; Zhao et al. 2014; Sun et al. 2023). Normally, sand rice forms monospecific populations on sand dunes and serves as a pioneer species for following vegetation restoration (Zhang et al. 2003; Liu et al. 2024). The above‐ground structures of sand rice, including leaves and stems, are covered with a high density of trichomes, which aid in water retention during dry conditions (Zhao et al. 2023). Additionally, nutrient resorption from senescent leaves enhances survival in nutrient‐poor soils. However, the subindividual variability in the redistribution of mineral elements within sand rice, particularly regarding how leaf age and trichomes influence this process, remains poorly understood. This study addresses two specific queries: (i) how do the concentrations of minerals vary across leaves of different ages in sand rice? and (ii) to what extent do trichomes influence these variations? By comparing trichome‐bearing wild‐type plants with a trichomeless mutant (Zhang et al. 2018), we aim to unravel the role of trichomes in nutrient allocation and desert adaptation. Our findings will advance understanding of resource allocation strategies in arid‐adapted plants, informing sustainable management practices for desert ecosystems.

## 2 Material and Methods

### 2.1 Plant Growth and Materials

The wild‐type plant was obtained from the Shapotou Station (104°57′E, 37°27′N, 1250 m above sea level) in the Tengger desert, Ningxia, China, and was named SPT in this study. The *Agriophyllum squarrosum trichomeless1* (*astcl1*) mutant was identified from the ethyl methanesulfonate (EMS) mutagenesis library derived from the wild type SPT (Zhang et al. 2018). Seeds from wild‐type (SPT) and mutant (*astcl1*) were sown on moistened filter papers within Petri dishes (10 cm diameter). Subsequently, the dishes were incubated in the dark in a growth chamber (RDN‐2600, Yanghui, China) for 2–3 days at a diurnal temperature regime of 30°C/20°C (14 h/10 h) to promote germination. After germination, seedlings were transplanted into pots (33 × 33 cm) filled with sand from the Shapotou Station. Under laboratory conditions, they were grown with a 14‐h light/10‐h dark photoperiod, maintaining a light intensity of approximately 150 μmol/m^2^/s. The pots were watered once a week with 500–600 mL of distilled water. These growth conditions were maintained for 3 months (from May 21, 2023 to August 21, 2023).

We observed trichome density on adaxial (upper) and abaxial (lower) surfaces of SPT leaves on the top of plants at two developmental stages: young (40‐day‐old) and old (89‐day‐old) (Ran et al. 2024). We then tested the adhesion strength of the trichomes in the laboratory by gently brushing both sides of the leaves with a water‐soaked calligraphy brush. Leaf age was determined using a position‐to‐age conversion method, as established in studies on other plant species (Wu et al. 2016). Specifically, the top, middle, and bottom leaves represent three distinct leaf ages. For the sample collection, three individual plants were selected from SPT and *astcl1* each, and from each plant, one leaf was randomly selected from the top, middle, and bottom parts. The leaves were then oven‐dried overnight at 85°C (DHG‐9203A, Yiheng, China), and the middle sections of dried materials were mounted on sterile petri dishes. Six target positions on these sections were analyzed using a Laser Ablation Inductively Coupled Plasma Mass Spectrometry (LA‐ICP‐MS) system for elemental profiling. The six target positions were selected from two rows perpendicular to the central vein, with three points on each side.

### 2.2 LA‐ICP‐MS Analysis

The LA‐ICP‐MS analysis was conducted using a laser ablation system (NWR213, ESI, USA) interfaced with a double focusing high resolution Element II ICP‐MS (ICAP‐TQ, Thermo Fisher Scientific, USA). The NWR213 employs an Nd:YAG solid‐state laser with a wavelength of 213 nm for element mapping measurements. All elemental signals were captured using the Element II ICP‐MS system. The LA‐ICP‐MS system was optimized for sensitivity before sample analysis using the NIST SRM612 glass reference material. Instrumental settings were as follows: the LA‐ICP‐MS unit was operated at 40% of maximum energy, with a spot size of 4 μm, laser fluence of 18.28 J/cm^2^, and scan time of 47 s, utilizing helium as the collision gas.

### 2.3 Multivariate Analysis

Non‐metric multidimensional scaling (NMDS) and principal coordinates analysis (PCoA) were performed in R (version 4.3.2) using the *vegan* and *ggalt* packages, respectively (Oksanen et al. 2024). Data visualization used *ggplot2* (Wickham 2016). Welch's ANOVA and Kruskal‐Wallis tests compared elemental concentrations across genotypes and leaf ages. Homogeneity of variance was assessed via Levene's test.

In order to elucidate the key factors contributing to the variance in elemental profiles, we performed a principal component analysis (PCA) to ascertain the most influential principal components using the *Z*‐score standardized data. The significance of each element's contribution was subsequently assessed by examining the calculated factor loadings. The PCA was conducted using the *FactoMineR* and *factoextra* R packages (Lê et al. 2008; Kassambara and Mundt 2020).

### 2.4 Violin Plot Generation

To show the individual elemental composition between SPT and *astcl1* plants at different leaf stages, violin plots were generated using the tools available on the website https://www.xiantaozi.com/products.

### 2.5 Regression Modeling

Multiple regression models were constructed to examine the effects of genotype and leaf age on each elemental profile. Prior to modeling, all predictor variables were standardized using log10 transformation. Four models were employed: Model 1 (*Y* = β0 + βg × Genotype), Model 2 (*Y* = β0 + βp × Position), Model 3 (*Y* = β0 + βg × Genotype + βp × Position), and Model 4 (*Y* = β0 + βg × Genotype + βp × Position + βgp × Genotype × Position). The selection of models was based on AIC (Akaike Information Criterion), with the minimum AIC value. In cases where multiple models had similar AIC values (ΔAIC < 2), the model with the fewest parameters was selected. Additionally, the relative importance of each variable on elemental profile was assessed by calculating various statistics, including parameter estimates, with the results listed in Table S1.

## 3 Results

### 3.1 Variation in Trichome Traits Across developmental stages

**FIGURE 1:**
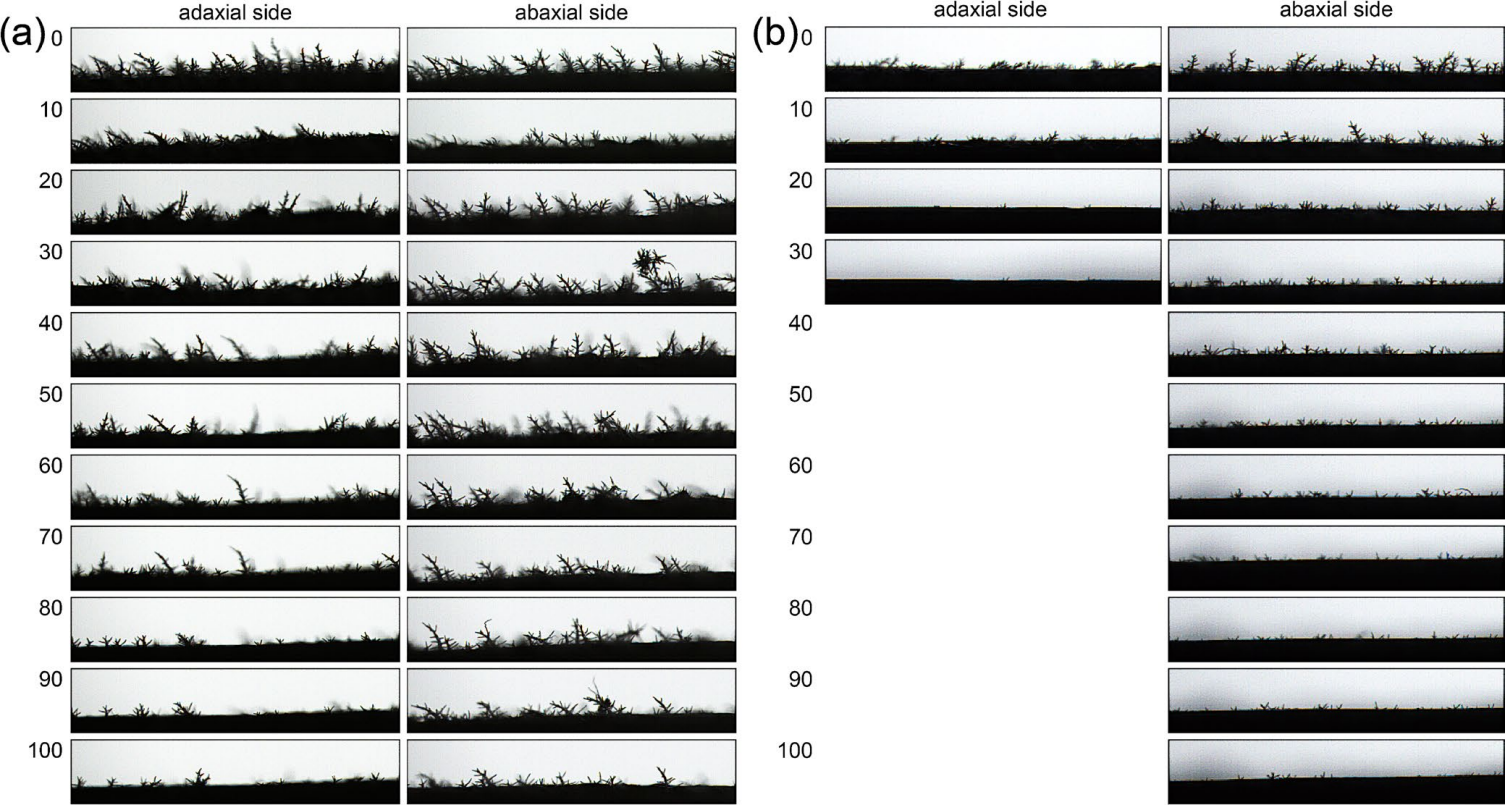
Trichome density on adaxial and abaxial surfaces of sand rice leaves. (a) Leaf trichome density at the young stage; (b) Leaf trichome density at the old stage. Numbers 0–100 represent the number of brushings with a writing brush after being dipped in water. The images were captured by a Contact Angle Goniometer (JC2000P, Powereach, China).

In this study, we found that trichome density on the abaxial (lower) surface of wild‐type (SPT) leaves exceeded that on the adaxial (upper) surface at both young and old developmental stages (Figure 1a,b). Trichome density declined obviously with leaf age on both leaf surfaces (Figure 1a,b). At young stage, a significant reduction in trichome density was observed on both leaf surfaces after 40 light brushings. Upon reaching 100 brushings, only a few trichomes remained, with a relatively higher concentration on the abaxial surface (Figure 1a). In contrast, for old stage, almost all trichomes on the adaxial leaf surface were removed after 30 gentle brushings, while the abaxial surface still had a few trichomes left even after 40–50 gentle brushings (Figure 1b).

### 3.2 Variations in Mineral Concentrations Within Sand Rice Leaves Between Wild Type and *Trichomeless* Mutant Across Different Leaf Ages

**FIGURE 2:**
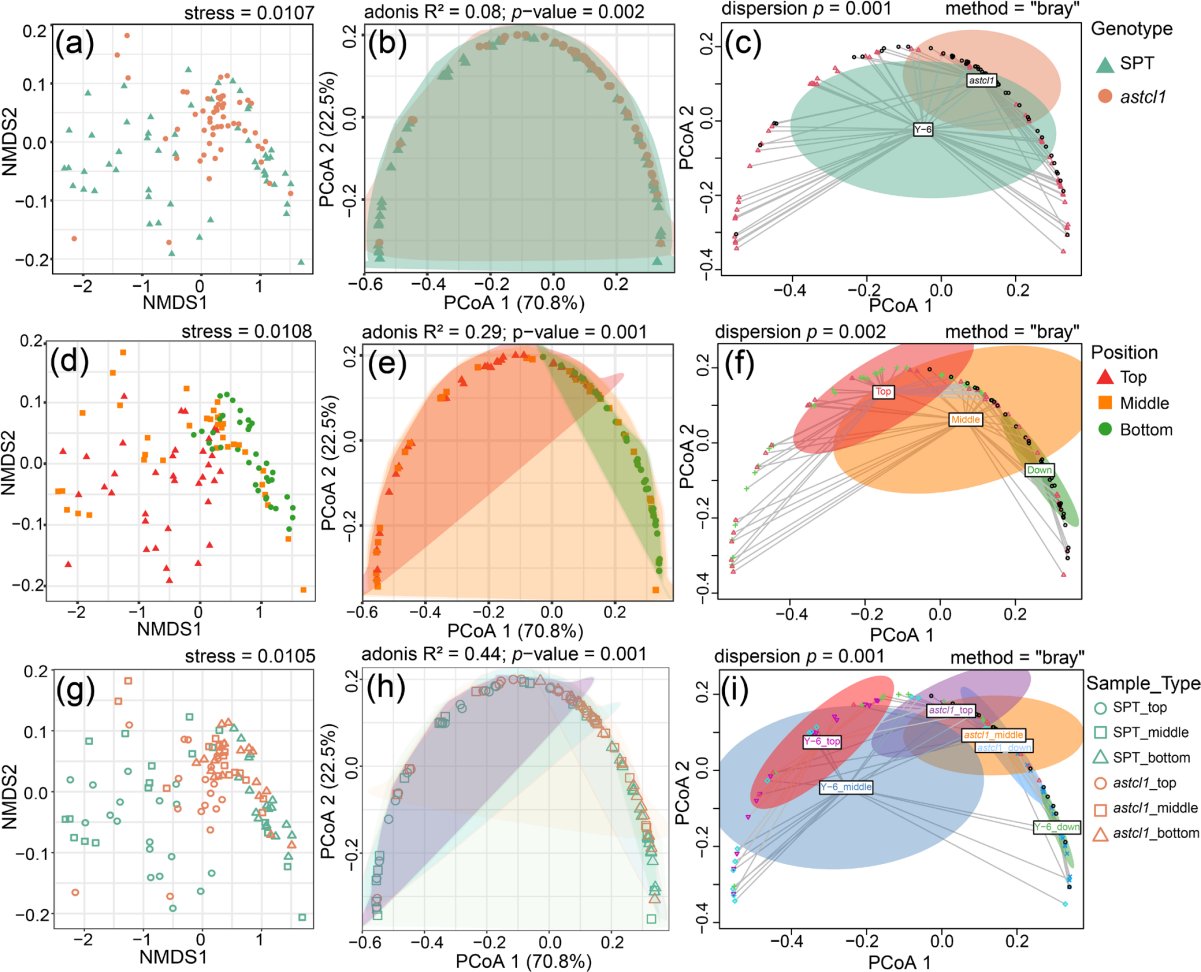
Analyses of the diversity in elemental profiles between the two genotypes and different leaf stages. (a, d, g) Non‐metric Multidimensional Scaling (NMDS) showing the distribution of samples from different groups based on Bray‐Curtis distance (stress = 0.01 for all plots); (b, e, h) Principal Coordinate Analysis (PCoA) plots of diversity between or among different groups. Principal components (PCoA) 1 and 2 account for 70.8% and 22.5% of the variance, respectively; (c, f, i) Dispersion analysis demonstrating the homogeneity of variances across groups using Levene's test (*p* < 0.01 for all groups).

LA‐ICP‐MS analysis successfully identified a total of 47 minerals, including 17 essential and 26 nonessential minerals (Additional File 1). The essential minerals can be further classified into macroelements and microelements. The macroelements are K, P, S, Ca, and Mg. The microelements are Fe, Mn, Zn, Cu, B, Mo, Cl, and Ni. Additionally, elements Na, Al, V, Co, Si, Se, Ga, and I are essential for some plants. Li, Rb, Cs, Be, Sr, Ba, Sc, Ti, Cr, Y, Nb, Ru, Rh, Pd, Ag, Hg, Ge, In, Sn, Sb, Tl, Pb, Bi, As, Te, and Br are non‐essential minerals. PCoA revealed genotype (SPT vs. *astcl1*), leaf age (top/middle/bottom), and their interactions explained 93.3% of elemental variance (PCoA1 + PCoA2; *p* < 0.01, Figure 2b,e,h). NMDS confirmed distinct clustering by genotype and age (stress = 0.011, Figure 2a,d,g). Despite clear clustering, significant dispersion within groups (*p* < 0.01, Levene's test) indicated subindividual variability contributes to elemental profiles (Figure 2c,f,j).

### 3.3 Leaf Age‐Dependent Mineral Composition Differences Between *Trichomeless* Mutant and Wild‐Type Plants

**FIGURE 3:**
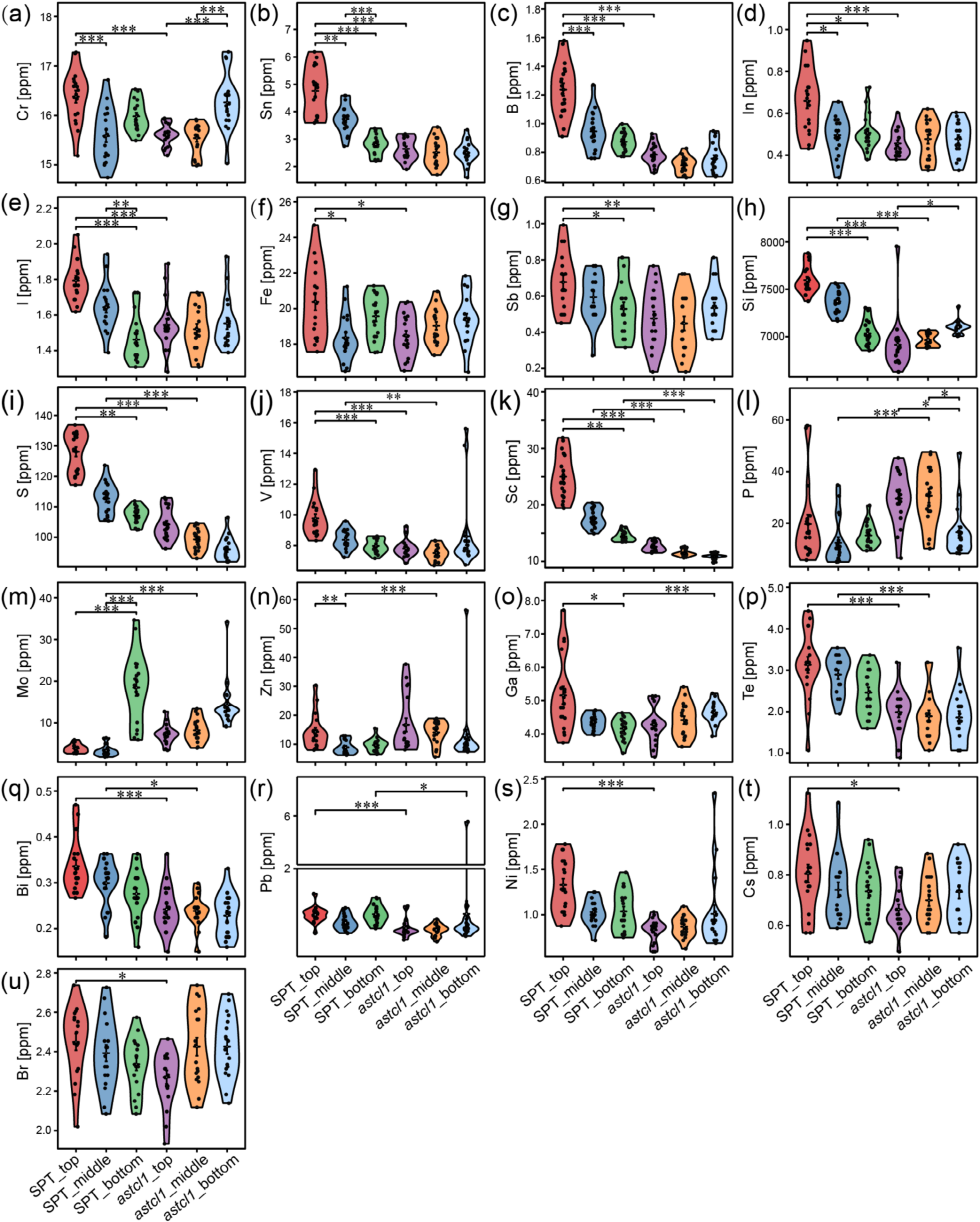
Violin plots of elemental levels in the top, middle, and bottom leaves of wild‐type (SPT) and mutant (*astcl1*) genotypes. (a–o) represent 15 elements that show significant differences in both the two genotypes and different leaf positions; (p–u) display six elements changed only in the two genotypes. Statistical significance levels are denoted by asterisks (*p < 0.05, **p < 0.01, ***p < 0.001).

Statistical analysis revealed significant differences in 33 of 47 leaf mineral elements across genotypes (wild‐type SPT vs. mutant *astcl1*), leaf positions (top, middle, and bottom), and their interactions (Figure 3 and Additional File 1). Fourteen elements showed no variation. A Venn diagram highlighted 15 elements with genotype‐ and leaf position‐dependent variation, encompassing macronutrients (P, S), micronutrients (Fe, Zn, B, Mo, V, Si, I, Ga), and non‐essential elements (Cr, Sn, In, Sb, Sc) (Figure 3 and Figure S1). Notably, Si and Cr varied significantly between genotypes and across leaf positions (Figure 3a,h). P varied within the mutant across leaf positions and between genotypes at the same position (Figure 3l). Top leaves showed differences in Sn, B, In, I, Fe, and Sb; middle leaves in S, V, Mo, and Zn; and bottom leaves in Sc and Ga (Figure 3b–g,i–k,m–o).

Six elements (Te, Bi, Pb, Ni, Cs, Br) differed solely between genotypes, with lower concentrations in *astcl1* (Figure 3p–u). Leaf position exclusively influenced 12 elements: Ca, Mg, Li, Mn, Ti, Cl, Hg, Cu, Sr, Y, Ba, and Na (Figure S2). Among these, Ca, Mg, Li, Mn, Ti, Cl, and Hg varied within the SPT across leaf positions, while the remaining elements differed in both genotypes.

### 3.4 Key Factors Affecting Elemental Concentration Differences Between the *Trichomeless* Mutant and Wild Type at Different Leaf Stages

**FIGURE 4:**
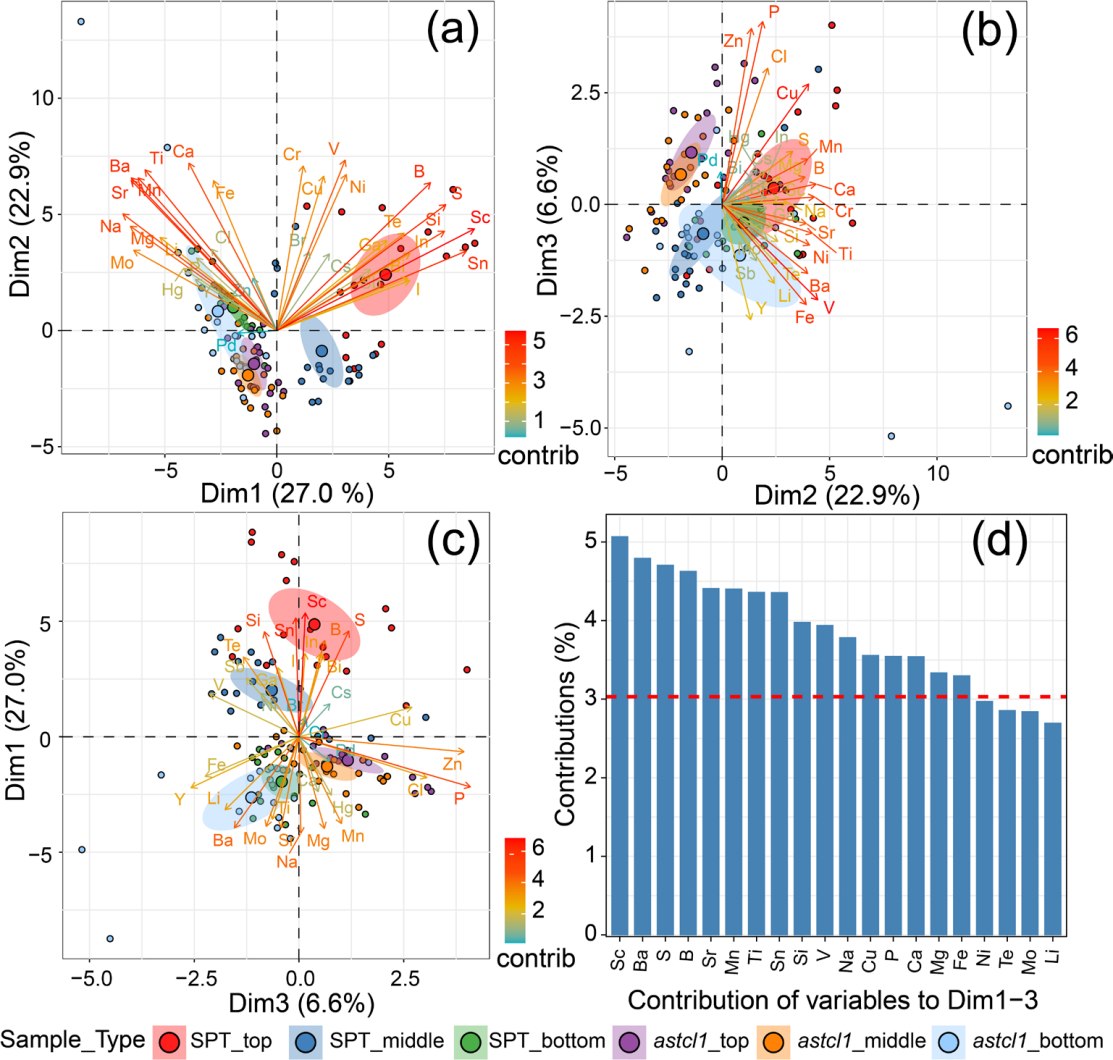
Visualization of the contributions of the 33 elemental concentration variables to the first three principal components (PCs). (a–c) Biplot visualizations depicting the correlations between the elemental variables and the first three PCs. Dim1, Dim2, and Dim3 present the first, second, and third principal components, respectively; (d) Bar plots showing the loadings (contribution) of individual elements on PC1‐3. The red line indicates the expected average contribution of each element.

To dissect the elemental concentration variations related to plant genotypes and leaf ages, we performed a Principal Component Analysis (PCA) on 33 elements. The first three principal components (PCs) accounted for 56.5% of total variance, highlighting the elemental concentration differences across genotypes, leaf ages, and their interactions (Figure S3 and Figure 4a–c). Notably, 16 elements, ranging from macronutrients (e.g., S, P, Ca, Mg) to micronutrients (e.g., B, Fe, Mn) and non‐essential elements (e.g., Ba, Sc, Ti), emerged as the most critical factors of the differences (Figure 4d).

Hierarchical and K‐means clustering further grouped elements based on correlations. Particularly, the concentrations of elements influenced by leaf position (e.g., Na, Mg, Mn, Ca) formed coherent clusters, indicating their age‐dependent roles (Figure S4). Similarly, elements affected by genotype and leaf position interactions (e.g., Si, Sn, B) clustered together, highlighting their shared regulatory pathways (Figure S4).

### 3.5 Impact of Trichome Defect Mutation and Leaf Age on Differences in Elemental Profiles

**FIGURE 5:**
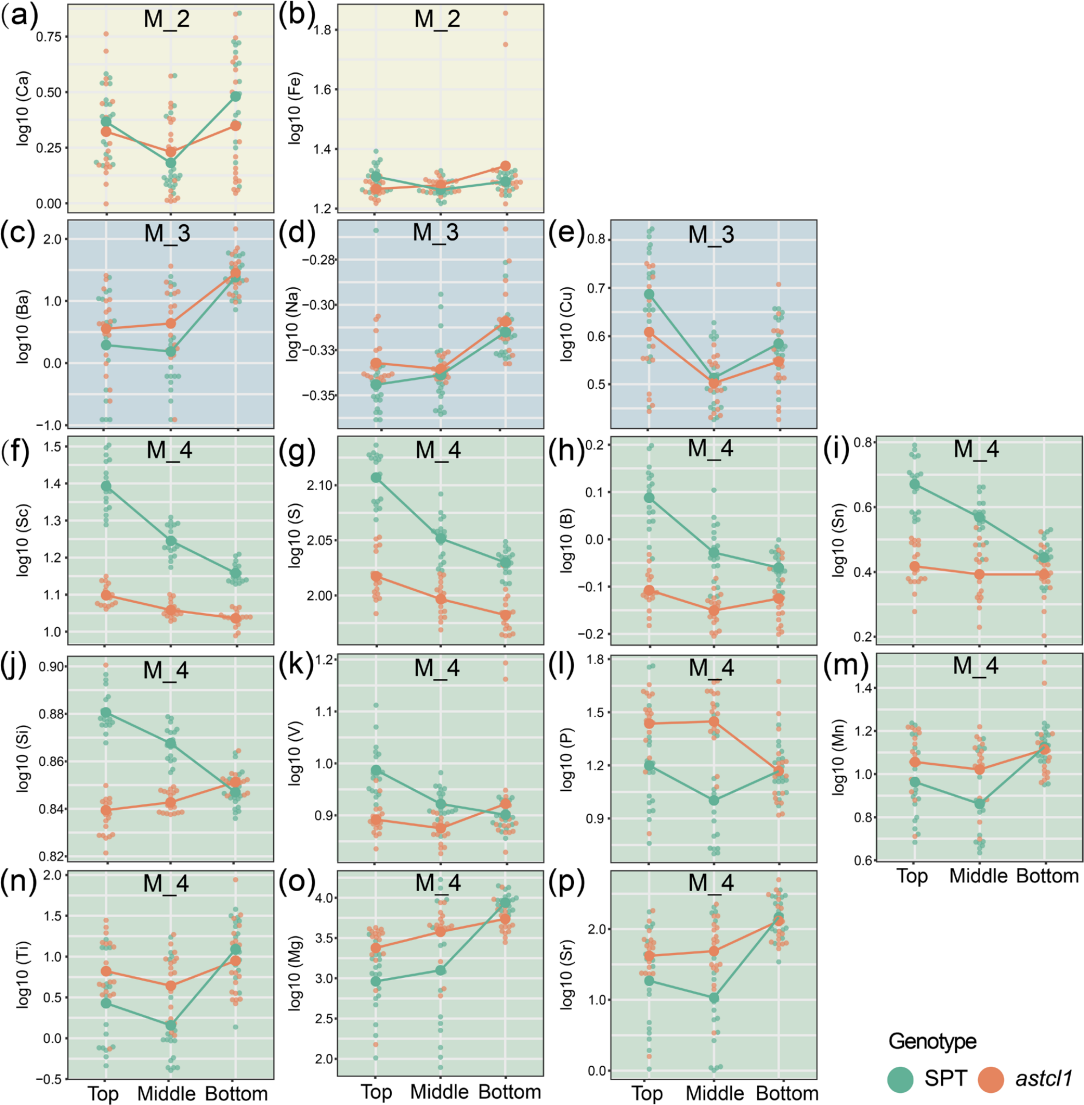
Evaluation of the influence of genotype, leaf position, and their interaction on elemental concentration variation through multivariate linear regression analysis. (a–p) depict the 16 elements that significantly contribute to the first three principal components.

Using multiple linear regression with log10‐transformed data, we tested four models to quantify genotype and leaf age effects. Model 1 (genotype‐only) significantly predicted variations in Te, Cs, and Pd (*R* ^2^ = 0.07–0.37, *p* < 0.05; Figure S5a,b,d and Table S1). Model 2 (leaf position‐only) explained Ca, Y, Li, and Fe concentrations (*R* ^2^ up to 0.23, *p* < 0.05; Figure 5a,b, Figure S5e,f and Table S1). Model 3 (genotype + leaf position) accounted for Na, Hg, Bi, Cu, Ba, and Zn (*R* ^2^ = 0.22–0.45, *p* < 0.05; Figure 5c–e, Figure S5g–i and Table S1). Seventeen elements (e.g., Sb, Mn, Ti, P, Mg, B) showed significant genotype‐only, leaf position‐only, and genotype × leaf age interactions in Model 4 (*p* < 0.05; Figure 5f–p, Figure S5j–m,q and Table  S1). Notably, Sc had the highest *R* ^2^ (0.91), emphasizing the interplay between genetic and developmental factors.

## 4 Discussion

### 4.1 Distinct Patterns of Mineral Concentrations at Different Leaf Stages

Mineral nutrients play a critical role for the normal growth and development of plants. In several plant species, minerals accumulation, both essential and nonessential, is influenced by developmental processes, including B in *A. thaliana* (Takano et al. 2001), P and K in *Medicago sativa* (Wang et al. 2014), P, K, Mg, and Ca in *Citrus sinensis* (Sanz et al. 1987), Cd in *Sedum alfredii* (Hu et al. 2019), Mn in *Grevillea meisneri* (Losfeld et al. 2015). In this study, we observed significantly subindividual differences in mineral profiles in the leaves at different stages in wild‐type sand rice (SPT) plants (Figure 2), consistent with previous findings (Watanabe et al. 2016; Ji et al. 2021). These different patterns are likely due to varying resource demands between young and old leaves. Younger, actively growing top leaves require more resources to support organ development and optimize light acclimation (Sultan 2000). Our observation showed that young leaves had significantly higher concentrations of macromineral S and microminerals such as Fe, Cu, Si, V, and B, as well as nonessential minerals like Sc and Sn (Figure 3 and Figure S2), probably linked to their elevated demands for growth and development processes, such as protein synthesis, photosynthesis, enzymatic functions, cell wall reinforcement, and defense mechanisms (Kusunoki 2011; Marschner 2012). In contrast, the macroelements Ca and Mg, microelements Na, and nonessential elements such as Ba, Sr, and Ti were found to be enriched predominantly in the middle or bottom leaves (Figure 3 and Figure S2), likely due to their roles in structural stability, ion balance, and other functions that are more critical in mature leaf tissues (Marschner 2012; Watanabe et al. 2016; Marler and Krishnapillai 2019). These within‐plant differences underscore the reallocation of resources, reflecting the complex and adaptive strategies employed by plants to meet the varying demands of different tissues at different stages of development.

### 4.2 The Influence of Trichomes on Minerals Profiles

Trichomes, as epidermal structures, are known to play critical roles in mineral uptake, storage, and detoxification (Agarie et al. 2007; Sarret et al. 2009; Marmiroli et al. 2004; Li et al. 2018; Abe 2019). Our investigation showed that the trichome density varies across developmental stages (Figure 1), a pattern consistent with other species, suggesting an ontogenetic defense strategy shift from mechanical prioritization in vegetative‐stage juvenile leaves to metabolic protection upon reproductive senescence (Schellmann and Hulskamp 2005; Nawab et al. 2011; Gago et al. 2016; Xu et al. 2019; Shahzad et al. 2021). Trichomeless (*astcl1*) mutants showed different mineral reallocation, particularly in young leaves (Figure 2). Most elements, except for Cu, Sr, Y, Ba, Na, P, and Cr, did not show significant differences in concentration among top, middle, and bottom leaves in *astcl1* plants, indicating impaired reallocation of these elements due to reduced trichomes (Figure 3 and Figure S2), highlighting the role of trichomes in the reallocation of minerals to meet the physiological demands. Among these elements, Ni is known to accumulate at the base of trichomes of the *Alyssum genus* (Ghasemi et al. 2009), and Pb accumulates in the prickle‐like trichomes of *Viola pricipis H. de Boiss* (Lei et al. 2008). However, other minerals such as Zn, Cu, Mn, Cd, Co, Tl, Se, Sr, and As, which are reported to accumulate in trichomes in other species (Arru et al. 2004; Li et al. 2005; Hopewell et al. 2021; Peco et al. 2020; Li et al. 2023), were not found in our study. This discrepancy might be due to the trichomes potentially being more waxy and having lower wettability compared to the cuticle in sand rice (Ran et al. 2025).

### 4.3 The Interplay Between *ASTCL1* Presence/Absence and Leaf Age in Mineral Composition

Moreover, the mineral composition is also affected by the interplay between leaf ages and the two genotypes (Figures 2, 3 and Figure S2). Sixteen key elements including S, P, Ca, Mg, B, Fe, Mn, Ba, Sc, and Ti, which mainly affect variations based on leaf age, genotype, and their interactions (Figures 4, 5), are clustered into five distinct groups according to their correlation coefficients (Figure S4). Among these, Ca, Sr, and Ba are clustered together, likely due to shared transport mechanisms (Watanabe et al. 2016). These findings indicate developmental and genetic coordination of mineral uptake, potentially through common regulatory pathways.

In summary, our findings reveal the intricate relationship between leaf age, trichomeless gene presence/absence, and mineral compositions in sand rice plants. These insights could be pivotal for understanding how plants manage mineral resources and for developing strategies to improve nutrient use efficiency. Furthermore, the significant dispersion within groups, indicating diversity and heterogeneity (*p* < 0.01), may be attributed to the sampling conducted from six distinct positions on the central cross‐section of a single leaf, leading to an uneven distribution of variance, further emphasizing the importance of studying the patterns of mineral concentrations within plants.

## 5 Conclusions

Changes in leaf functional traits, such as trichome density and nutrient composition, with leaf age highlight subindividual variation within the plant, reflecting plant life history strategies and the allocation of leaf resources. In this study, we examined the mineral element composition in sand rice leaves with varying trichome characteristics due to genetic differences and leaf ages, assessing 47 distinct minerals. We observed significant variability in elemental composition related to genotype, leaf age, and their interactions. Out of the 47 minerals studied, 33 showed significant influence by these factors. Sixteen critical elements (e.g., S, P, Ca, Mg, Fe, Mn) were identified as the primary factors of the observed differences in elemental composition. Multiple linear regression further assessed the impact of these factors. By identifying key elements influenced by genotype and developmental stage, we have contributed to understand the underlying mechanisms of resource allocation strategies within individual plants, offering potential pathways for plant adaptation in harsh environments.

## Author Contributions

**Ruilan Ran:** formal analysis (lead), investigation (equal), validation (equal), visualization (lead). **Xiaoyun Cui:** conceptualization (equal), funding acquisition (equal), investigation (equal), validation (equal), writing – original draft (equal), writing – review and editing (equal). **Xin Zhao:** investigation (equal), validation (equal). **Yingxue Zhao:** investigation (equal), validation (equal). **Caixia Zhang:** investigation (equal), validation (equal). **Pengshan Zhao:** conceptualization (equal), funding acquisition (equal), writing – original draft (equal), writing – review and editing (equal).

## Conflicts of Interest

The authors declare no conflicts of interest.

## Supporting information


**Additional File 1:** Raw data of Elemental profile analyzed using LA‐ICP‐MS.

**Figure S1:** A Venn diagram illustrating the overlap of minerals that were affected by genotypes and leaf ages. The diagram has three circles: a blue circle representing genotype, an orange circle representing leaf age, and a pink circle representing the overlap of the two.

**Figure S2:** Violin plots depicting the patterns of selected elemental concentrations exhibiting significant differences among different positions within the same genotypes (wild‐type SPT and mutant *astc1*). Statistical significance levels are denoted by asterisks (*p < 0.05, **p < 0.01, ***p < 0.001).

**Figure S3:** Parallel analysis of 33 elemental concentration variables. (a) Scree plot based on parallel analysis. Principal component (PC) eigenvalues are plotted on the y axis. Note that the PC simulated data and PC resampled data lines overlap. (b) Explained variance percentage. The x‐axis shows the principal components (dimensions), totaling 10 in this case. The y‐axis represents the percentage of variance explained by each principal component.

**Figure S4:** Correlogram with 16 element concentrations in sand rice leaves between the two genotypes at different leaf stages. The heatmap visualizes the Pearson correlation coefficients between the concentrations of 16 elements across genotypes and leaf positions. Positive and negative correlations are shown in blue and red colors, respectively, with the intensity indicating the magnitude of the correlation. Correlation coefficients with *p* < 0.001 are considered statistically significant. The heatmap was generated using the *corrplot* R package. Blocks indicate clusters of elements based on unassisted hierarchical clustering. Numbers of clusters are defined based on K‐means clustering.

**Figure S5:** Evaluation of the influence of genotype, leaf position, and their interaction on the variation in concentrations of the remaining 17 elements through multivariate linear regression analysis.

**Table S1:** Summary of multivariate linear regression analysis parameters for predicting the impact of the two genotypes and different leaf positions on elemental concentration variability.

## Data Availability

All data supporting the findings of this study are available in the main text and in the Supporting Information (Additional File 1) provided online.
