# Supplementary figures and images for: Within‐Plant Leaf Maturity and Trichome Density Variation Shape the Elemental Composition Divergence in Desert Plant Sand Rice (Agriophyllum squarrosum)
Source: Ecol Evol. 2025 Jun 12;15(6):e71542. doi: 10.1002/ece3.71542 (PMC12162362; doi:10.1002/ece3.71542)

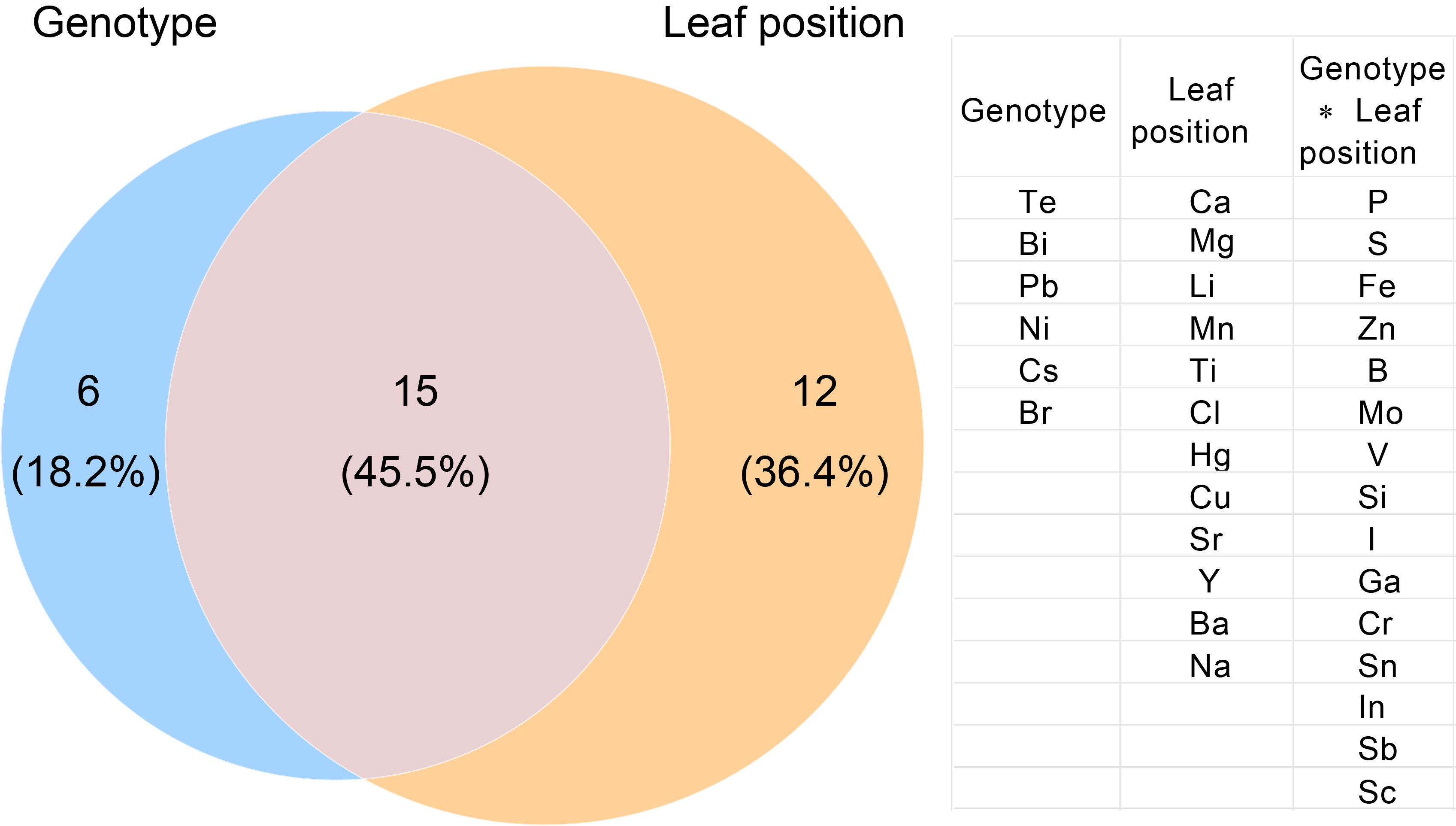

Supplement: Supplementary file 2 — Figure S1: A Venn diagram illustrating the overlap of minerals that were affected by genotypes and leaf ages. The diagram has three circles: a blue circle representing genotype, an orange circle representing leaf age, and a pink circle representing the overlap of the two. [file ECE3-15-e71542-s004.png]

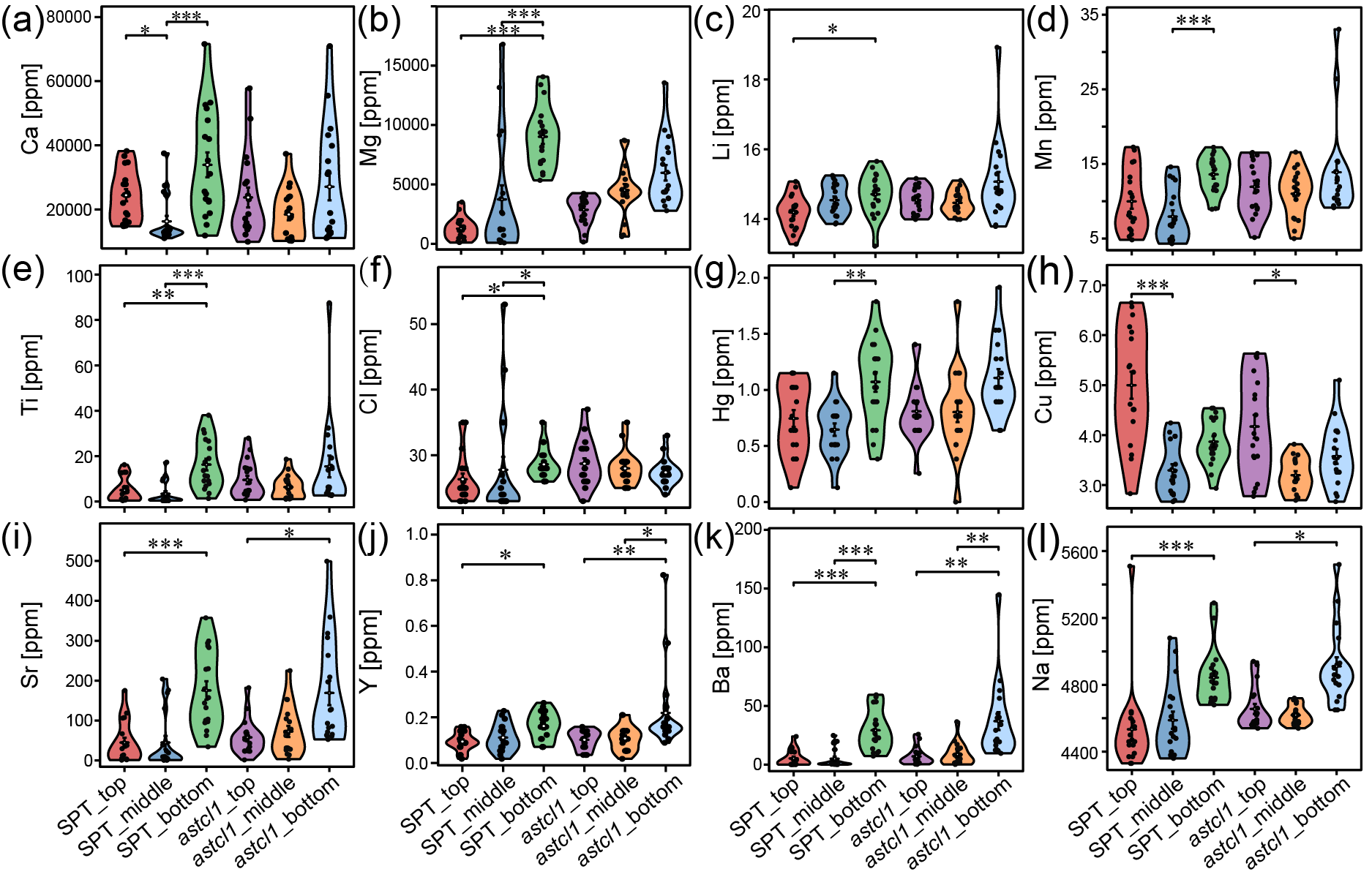

Supplement: Supplementary file 3 — Figure S2: Violin plots depicting the patterns of selected elemental concentrations exhibiting significant differences among different positions within the same genotypes (wild‐type SPT and mutant astc1). Statistical significance levels are denoted by asterisks (*p < 0.05, **p < 0.01, ***p < 0.001). [file ECE3-15-e71542-s003.png]

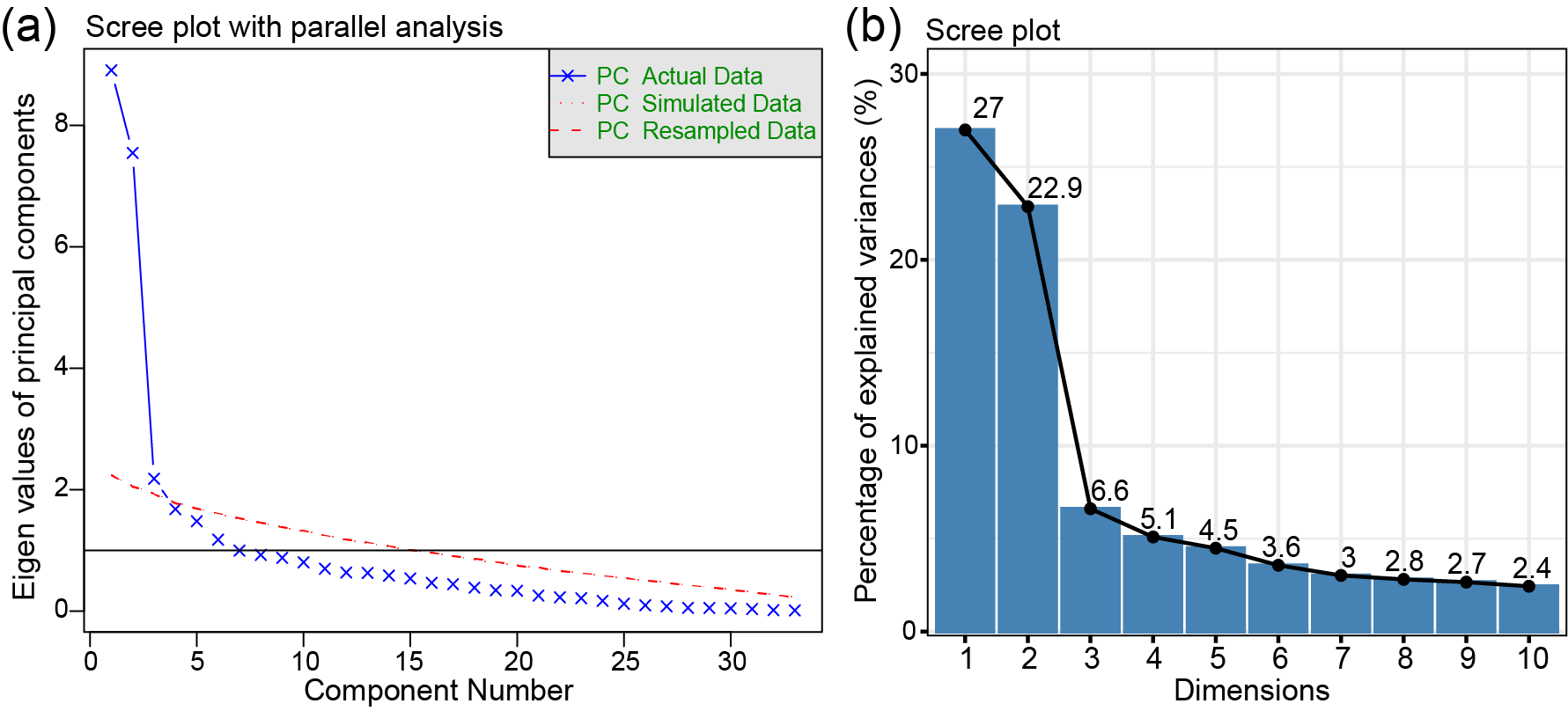

Supplement: Supplementary file 4 — Figure S3: Parallel analysis of 33 elemental concentration variables. (a) Scree plot based on parallel analysis. Principal component (PC) eigenvalues are plotted on the y axis. Note that the PC simulated data and PC resampled data lines overlap. (b) Explained variance percentage. The x‐axis shows the principal components (dimensions), totaling 10 in this case. The y‐axis represents the percentage of variance explained by each principal component. [file ECE3-15-e71542-s006.png]

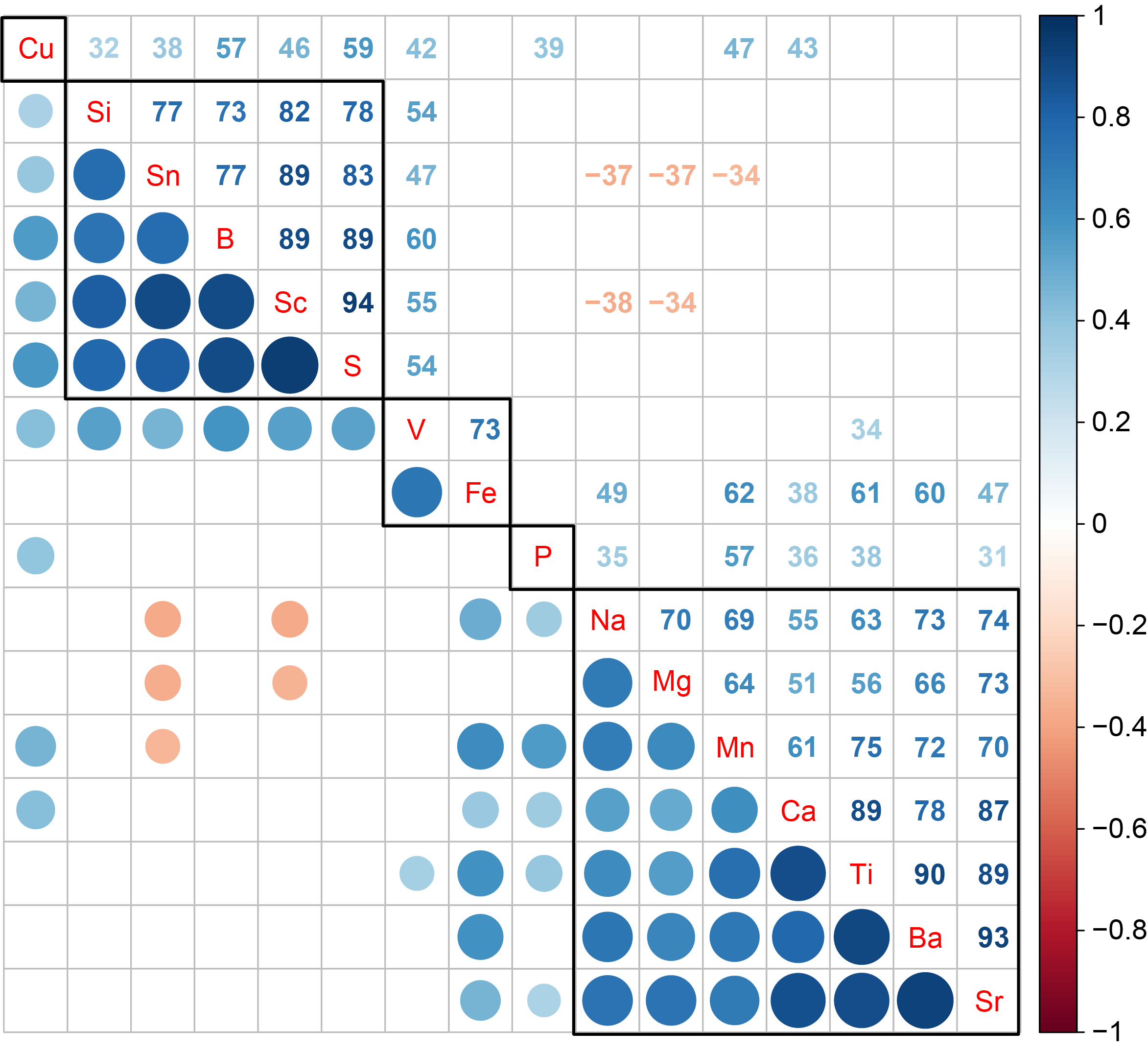

Supplement: Supplementary file 5 — Figure S4: Correlogram with 16 element concentrations in sand rice leaves between the two genotypes at different leaf stages. The heatmap visualizes the Pearson correlation coefficients between the concentrations of 16 elements across genotypes and leaf positions. Positive and negative correlations are shown in blue and red colors, respectively, with the intensity indicating the magnitude of the correlation. Correlation coefficients with p < 0.001 are considered statistically significant. The heatmap was generated using the corrplot R package. Blocks indicate clusters of elements based on unassisted hierarchical clustering. Numbers of clusters are defined based on K‐means clustering. [file ECE3-15-e71542-s002.png]

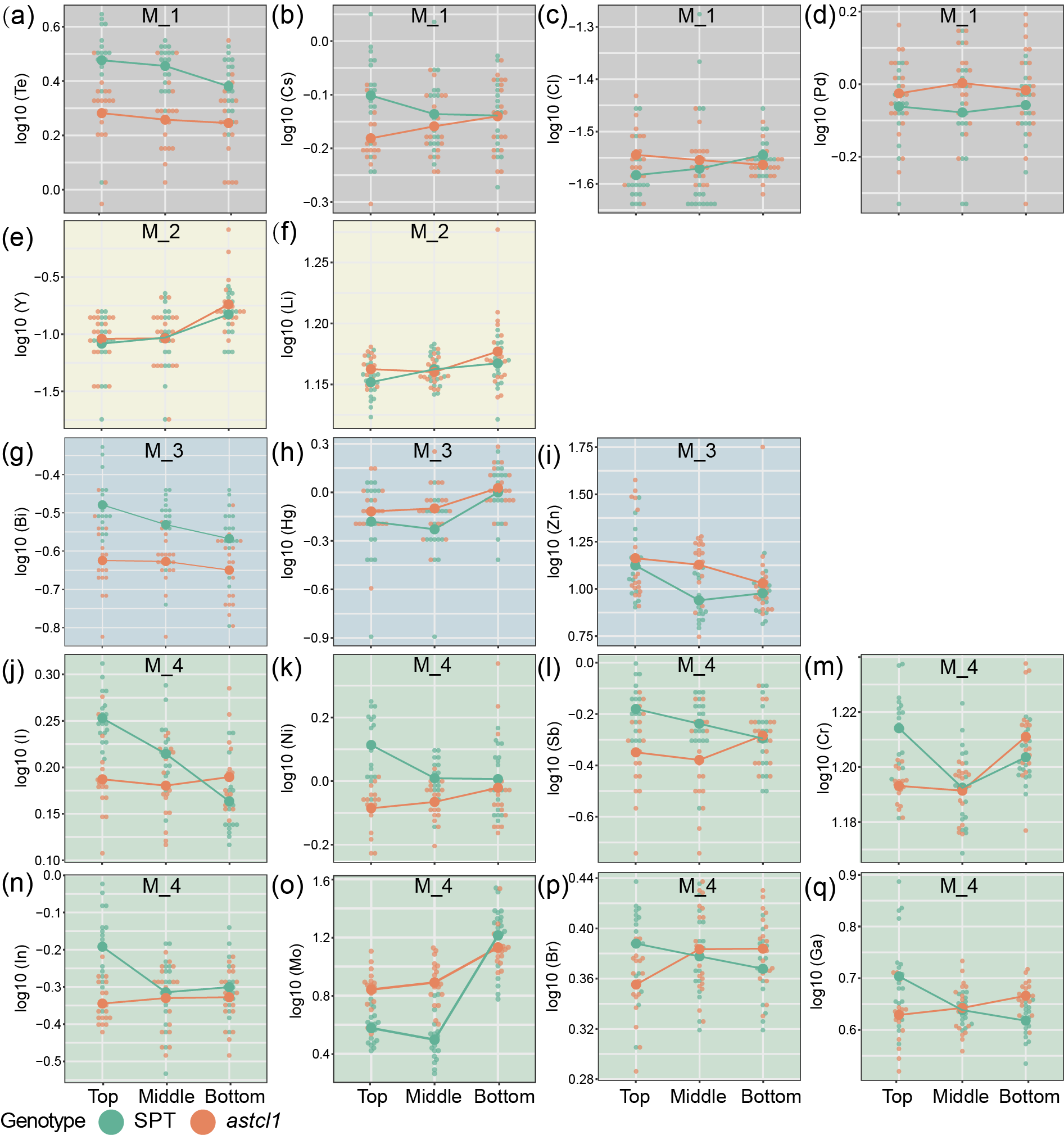

Supplement: Supplementary file 6 — Figure S5: Evaluation of the influence of genotype, leaf position, and their interaction on the variation in concentrations of the remaining 17 elements through multivariate linear regression analysis. [file ECE3-15-e71542-s007.png]
